# Supplementary material for: Identification of an Isoflavonoid Transporter Required for the Nodule Establishment of the Rhizobium-Fabaceae Symbiotic Interaction
Source: Front Plant Sci. 2021 Oct 22;12:758213. doi: 10.3389/fpls.2021.758213 (PMC8570342; doi:10.3389/fpls.2021.758213)
Supplement: Supplementary file 1 [file Data_Sheet_1.docx]

**Supplementary materials**

**
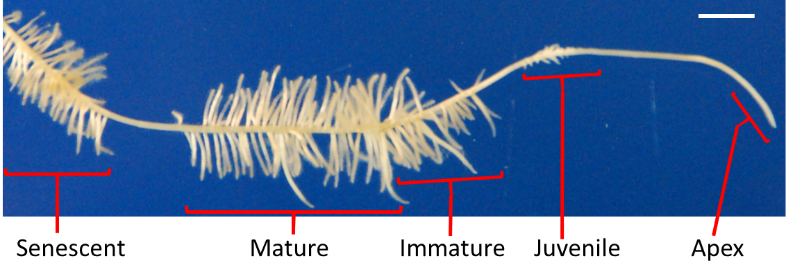
**

**Figure S1. Cluster roots of P-deficient white lupin**

Picture of a lateral root of a 4-week-old white lupin grown under P-deficient condition (+N-P). The different developing stages: Juvenile, Immature, Mature, Senescent cluster-root stage and Apex tissue [10 mm from the root tips] are highlighted and named. White scale bar = 10 mm.


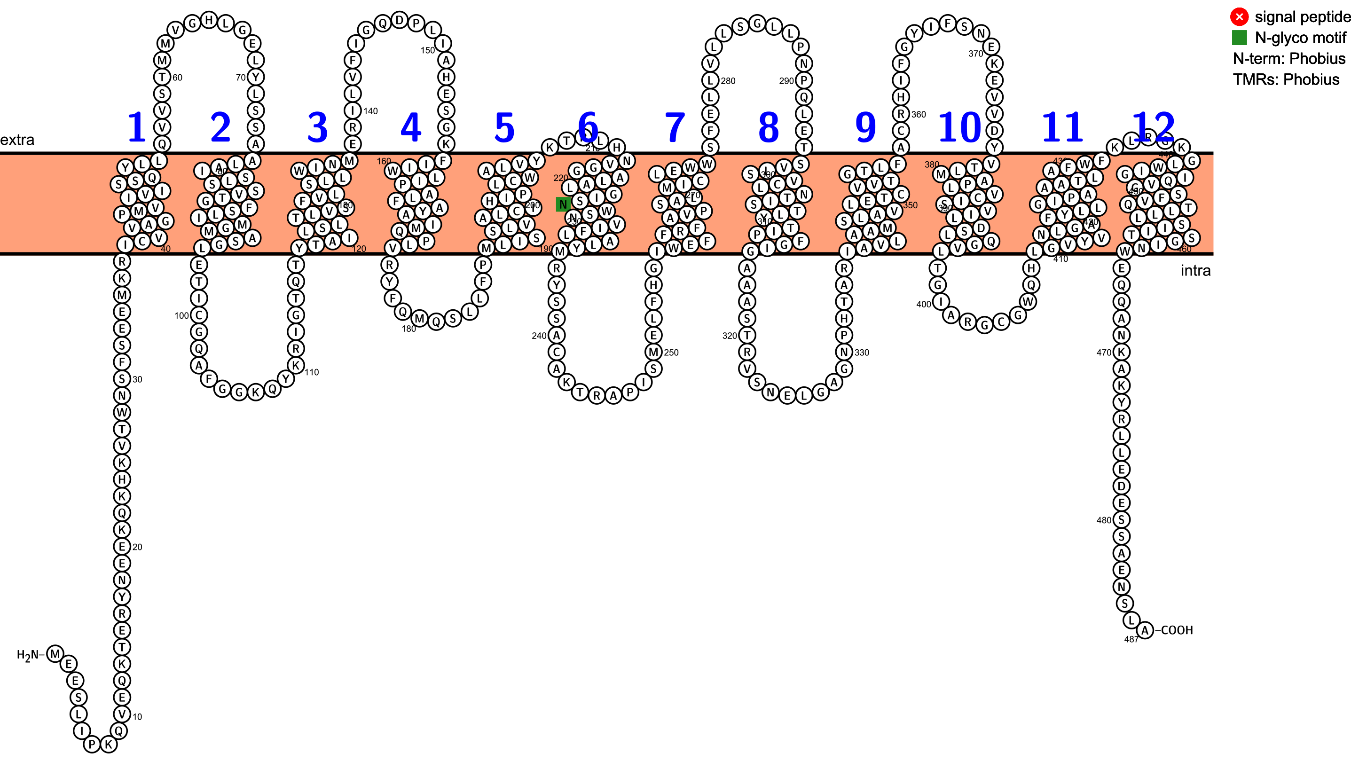


**Figure S2. Predicted membrane topology for LaMATE2 from white lupin**

The model was obtained by using the PROTTER program (Omasits *et al.,* 2014).


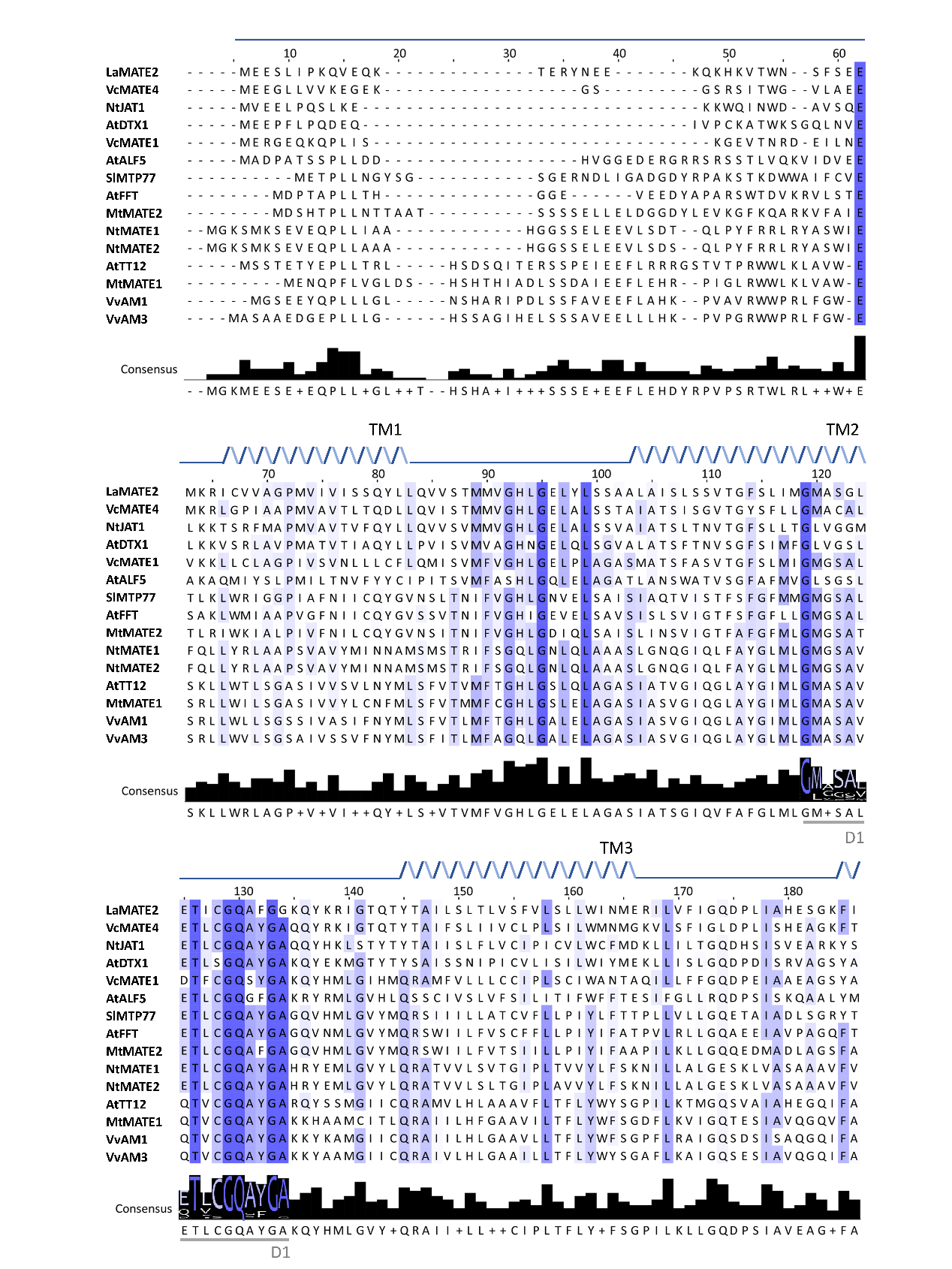


**
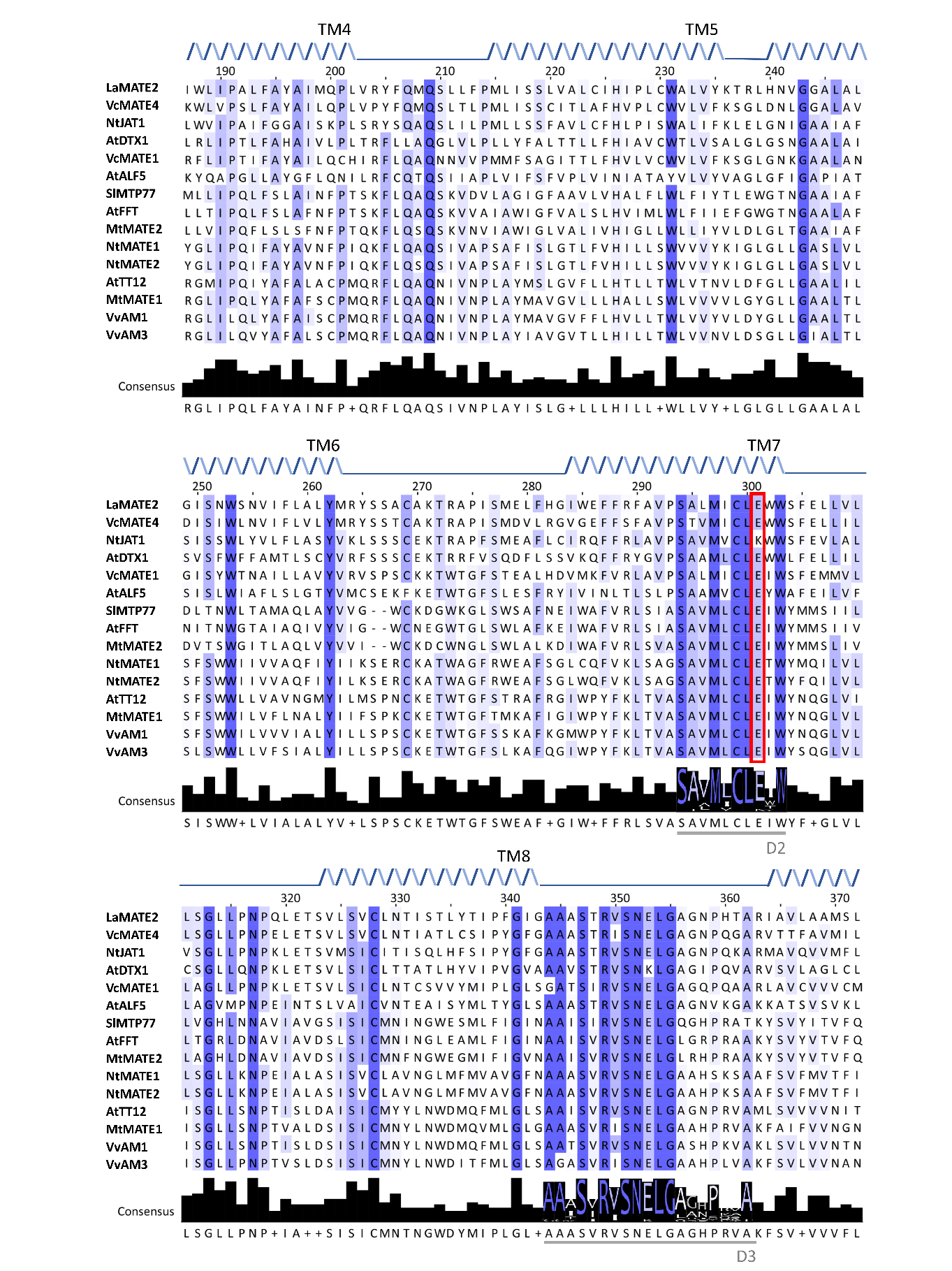
**


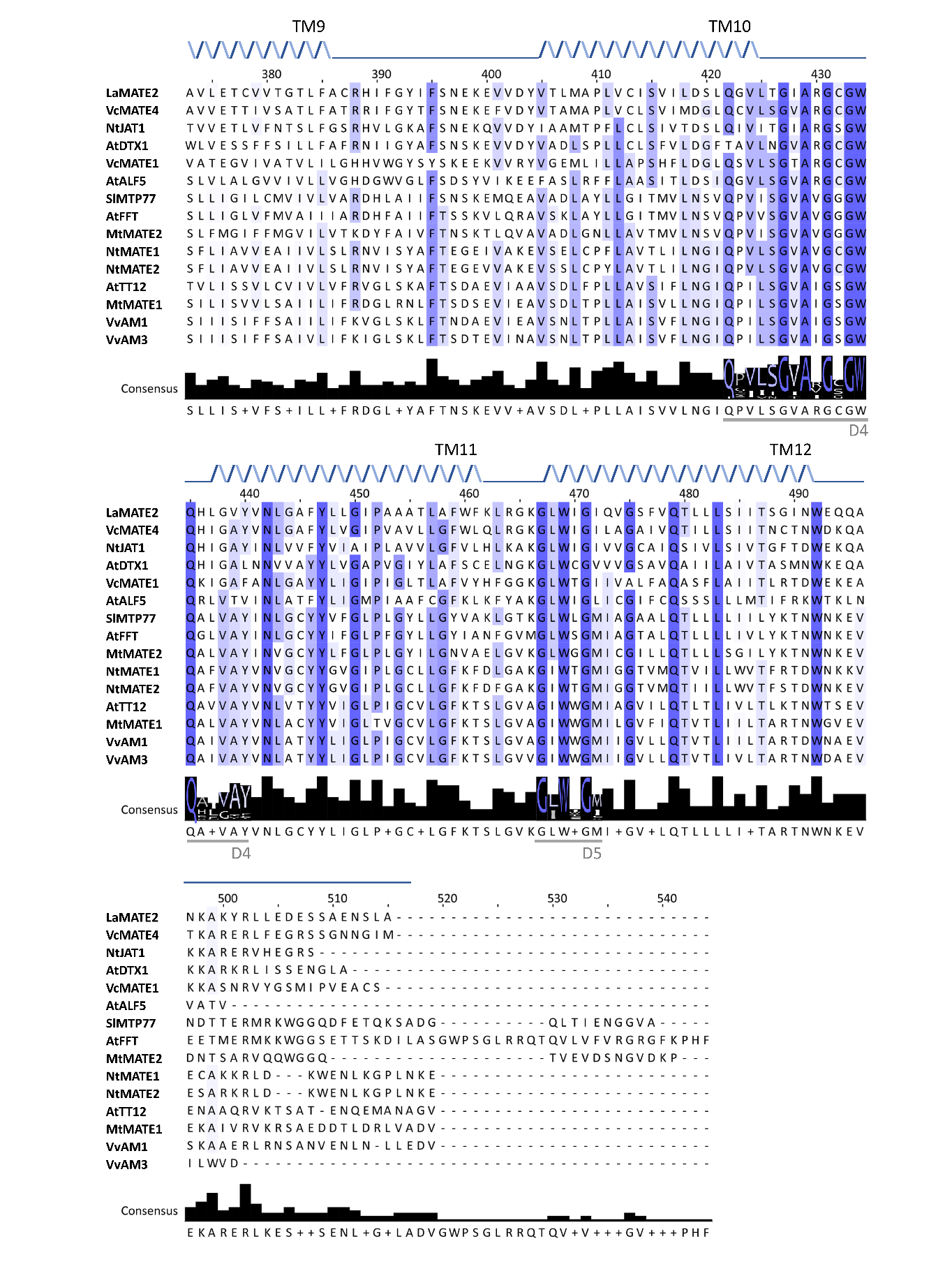


**Figure S3. Multiple sequence alignment of amino acid sequence of LaMATE2 with selected MATE transporter orthologs in higher plants**

Protein sequence alignment was performed using Clustal-WS using Jalview software version 2 (Waterhouse *et al.,* 2009). Amino acids with only highly conservative substitutions are highlighted in colour blue. Thin grey lines below the consensus sequence (D 1-5) indicate five short stretches of conservative amino acids reported for all 56 Arabidopsis MATE proteins. The red box indicates the residue E290 (TT12), constituting the cation-binding site in the pore. Twelve putative-transmembrane helical domains (TM 1-12) of LaMATE2 are indicated above the alignment


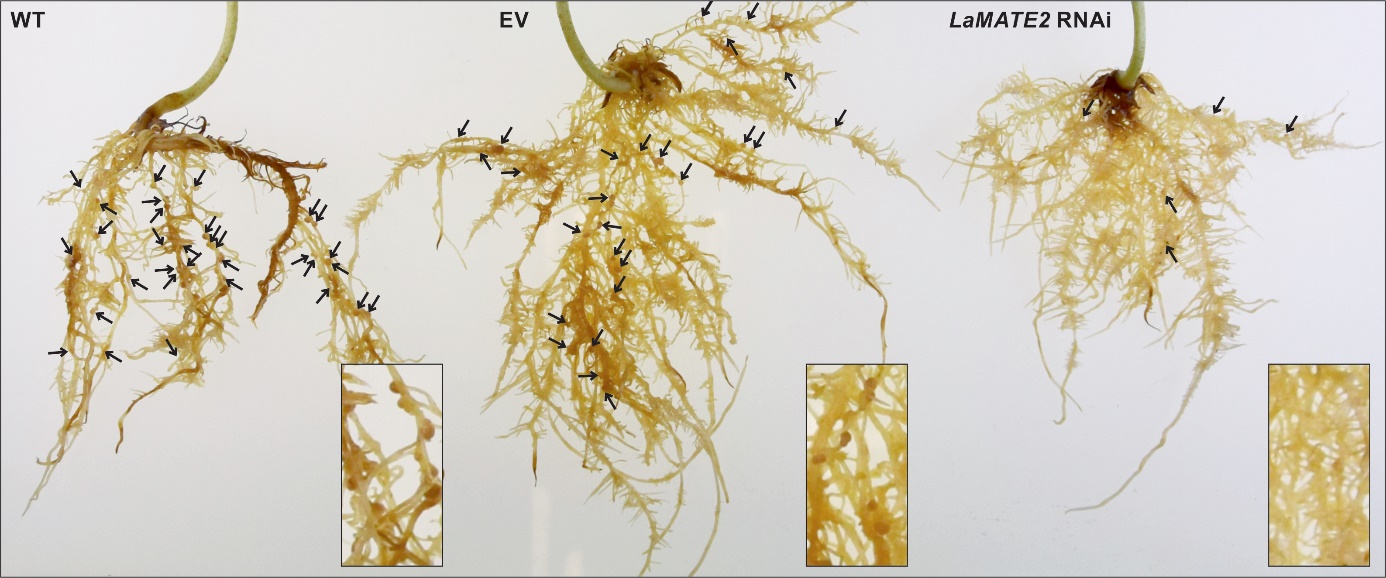


**Figure S4. Pictures of *LaMATE2*-silenced roots grown under N-deficient conditions**

Roots are shown in the following order: wild type, empty-vector-transformed roots and *LaMATE2*-RNAi-transformed roots. Nodule localizations are highlighted with arrows and close-up inserts are shown in the bottom right corners.


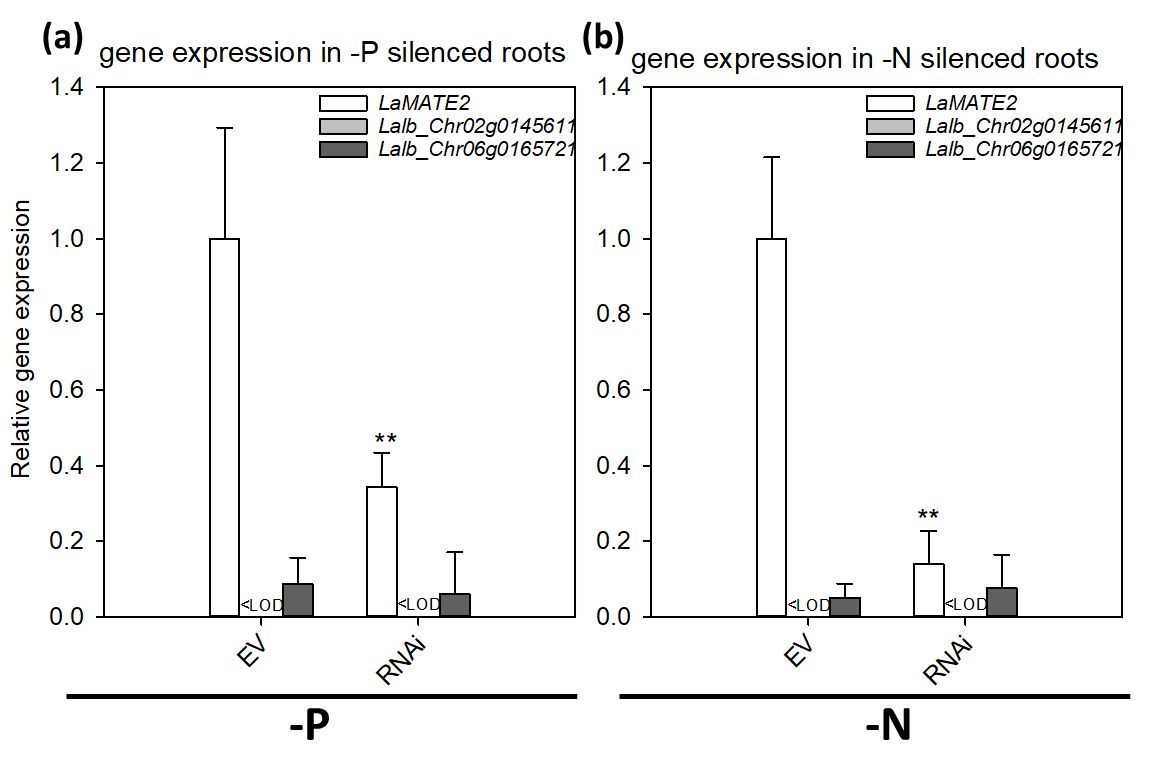


**Figure S5. Gene expression in the silencing experiments**

Relative expression of *LaMATE2* and the two closest homologues: *Lalb_Chr06g0165721* and *Lalb_Chr02g0145611* in P-deficient (**a**) and N-deficient (**b**) condition in pRedRoot::*LaMATE2* RNAi (RNAi) or empty-vector pRedRoot (EV) transformed roots. Expression data are expressed relative to *LaMATE2* expression in EV-transformed roots. Data are means+SD (* refers to statistically significant differences among the mean value of RNAi and EV, ANOVA Holm–Sidak, N=3, **P <0.01).

**Table S1.**

List of primers

|  | forward | reverse |
| --- | --- | --- |
| *LaMATE2* | cttgttttgctgtctgggct | tgcagcaccaattccaaagg |
| *Lalb_Chr06g0165721* | gtgaaacgatgaacatagaac | ctttgttgctgttgatgatg |
| *Lalb_Chr02g0145611* | agtgtcaacaccattgca | gatctaaccttgctgcag |
| *LaMATE2-GFP* | atagctagcatggaagagagtctaatacc | atacatgcctcatgctagactattttctgc |
| pNEV-*LaMATE2*ORF | atgcggccgcatggaagagagtctaatacc | agcggccgctcatgctagactattttctg |
| *LaMATE2* RNAi a | atccatggggcacgagatttctatct | gtgcatttaaatccacaaatagtttccagtc |
| *LaMATE2* RNAi b | atggatccccacaaatagtttccagtc | tgcactagtggcacgagatttctatct |
| *LaUbiquitin* | gcaccctagccgactacaac | ccggtaagggtcttgacaaa |
